# Impact of α-Globin Gene Expression and α-Globin Modifiers on the Phenotype of β-Thalassemia and Other Hemoglobinopathies: Implications for Patient Management

**DOI:** 10.3390/ijms25063400

**Published:** 2024-03-17

**Authors:** Joanne Traeger-Synodinos, Christina Vrettou, Christalena Sofocleous, Matteo Zurlo, Alessia Finotti, Roberto Gambari

**Affiliations:** 1 Laboratory of Medical Genetics, National and Kapodistrian University of Athens, St. Sophia’s Children’s Hospital, 11527 Athens, Greece; cvrettou@med.uoa.gr (C.V.); csofokl@med.uoa.gr (C.S.); 2 Department of Life Sciences and Biotechnology, 40124 Ferrara, Italy; matteo.zurlo@unife.it (M.Z.); alessia.finotti@unife.it (A.F.); 3 Center “Chiara Gemmo and Elio Zago” for the Research on Thalassemia, Department of Life Sciences and Biotechnology, University of Ferrara, 44121 Ferrara, Italy

**Keywords:** β-thalassemia, sickle cell disease, α-globin, α-globin-stabilizing protein, autophagy

## Abstract

In this short review, we presented and discussed studies on the expression of globin genes in β-thalassemia, focusing on the impact of α-globin gene expression and α-globin modifiers on the phenotype and clinical severity of β-thalassemia. We first discussed the impact of the excess of free α-globin on the phenotype of β-thalassemia. We then reviewed studies focusing on the expression of α-globin-stabilizing protein (AHSP), as a potential strategy of counteracting the effects of the excess of free α-globin on erythroid cells. Alternative processes controlling α-globin excess were also considered, including the activation of autophagy by β-thalassemia erythroid cells. Altogether, the studies reviewed herein are expected to have a potential impact on the management of patients with β-thalassemia and other hemoglobinopathies for which reduction in α-globin excess is clinically beneficial.

## 1. Introduction

A major factor influencing the phenotypic expression of human hemoglobinopathies is the degree of globin chain imbalance. Most notably, the excess of α-globin chains tends to be more deleterious to the erythrocyte, compared to excesses of other globin chains. Free α-globin chains cannot form homo-tetramers, as they are highly unstable and tend to aggregate, forming insoluble precipitates in the cell [1]. The reactive oxygen species (ROS) formed as a result, underlie red cell membrane damage, ultimately causing premature cell death, observed as ineffective erythropoiesis. This subsequently triggers apoptosis, causing the release of immature and nucleated erythrocytes into the circulation. In this review, we present and discuss studies on the normal expression of α-globin genes, as well as in individuals affected by hematopoietic diseases (such as β-thalassemia), focusing on the impact of altered α-globin gene expression and α-globin modifiers on phenotype and clinical severity of the diseases. We first discuss the impact of free α-globin excess on the phenotype of β-thalassemia. We then review studies focusing on the expression of α-globin-stabilizing protein as a strategy of erythroid cells to counteract the effects of free α-globin excess. Alternative processes controlling α-globin excess have also been considered, including the activation of autophagy by β-thalassemia erythroid cells.

## 2. Genetics, Pathophysiology, and Clinical Picture of Beta-Thalassemia

The hereditary β-thalassemia syndromes are primarily caused by mutations of the b-globin gene (HBB) [1,2,3]. Interestingly, about 20 HBB gene variants (of the more than 350 variants reported so far) account for more than 80% of the β-thalassemia variants worldwide [4,5]. These variants cause a large variety of phenotype alterations, leading to low or absent production of the β-globin polypeptide chain and, hence adult hemoglobin (HbA, α_2_β_2_) [1,2]. Clinically, TDT (transfusion-dependent thalassemia) and NTDT (non-transfusion-dependent thalassemia) refer to two major different phenotypes, the most severe of them (TDT) requiring regular blood transfusion and chelation therapy to maintain life. A key factor influencing the clinical severity in β-thalassemia patients and the expression of either TDT or NTDT is related to the presence of an excess of free β-globin chains in β-thalassemia erythroid cells [1,2]. The α-globin subunits tend to aggregate and then precipitate in the erythroid cells, causing deleterious effects, overall cytotoxicity and ineffective erythropoiesis. As a result, there is a general consensus on the existence of an interplay between β-globin gene expression, the amount of α-globin production, and the severity of clinical expression in β-thalassemia patients. This is the major focus of this short review.

## 3. Alpha-Globin Gene Cluster and Alpha-Globin Biosynthesis

The level of α-globin genes expression is strongly dependent on variants in the α-globin gene cluster, which may either increase or, more commonly, decrease the synthesis of the α-globin polypeptide chains, with potential consequences on the hematological and clinical phenotype of both β-thalassemia and sickle cell disease.

**Figure 1:**
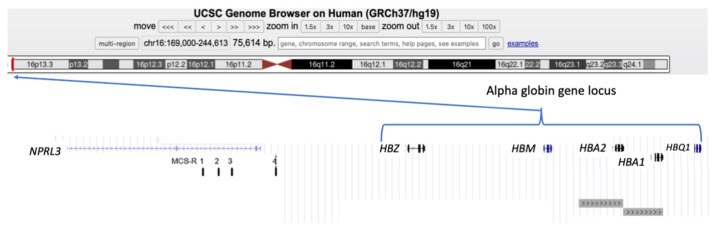
The alpha-globin gene cluster. Location of genes in part of the p13.3 region of chromosome 16, including the alpha-globin gene cluster. From telomere towards centromere (5′ to 3′), the genes are as follows: *NPLR3*, *HBZ*, *HBM*, *HBA2*, *HBA1*, and *HBQ1*. The extent of the homologous sequences surrounding *HBA1* and *HBA2* are depicted as grey boxes at the bottom of the diagram. The locations of the four MCS-R sequences 1–4 are also depicted under *NPRL3* (Adapted from the UCSC Genome Browser tool http://genome.ucsc.edu, (accessed on 11 February 2024).

Alpha-globin is the product of two almost identical α-globin genes, *HBA1* and *HBA2* (previously known as α1 and α2), located on the short arm of chromosome 16 (Chr16p13.3, GenBank NG_000006). As with all human globin genes, the α-globin genes have three exons and two introns (or intervening sequences, IVSs), which interrupt the coding sequence between codons 31/32 and codons 99/100. The length of *HBA1* and *HBA2* are 842 and 834 nucleotides (base pairs, bps), respectively, and they are positioned on the forward strand of chromosome 16p, meaning that they are expressed (transcribed) from the telomeric towards centromeric direction. The coding sequences (exons) of *HBA1* and *HBA2* are identical. However, the two genes differ slightly in their 5′ untranslated regions (UTRs) and their introns, and there are significant differences in their 3′UTRs. Furthermore, the duplicated α-globin genes are both embedded within larger homologous sequences, which play a role in the production of the two most common α^+^ thalassemia deletions (see Figure 1 and Section 3) [6].

The human α-globin cluster is located about 150 kb from the telomere of the short arm of chromosome 16, in a region of the genome that is GC-rich (around 54%), contains many Alu-repeat sequences and many genes. In fact, the α-globin gene cluster is surrounded by widely expressed genes and, furthermore, the upstream regulatory elements (including HS-40) lie within the introns of the adjacent housekeeping gene, *NPRL3* [6,7].

There are conserved sequences motifs within the 5′ promoter regions of the *HBA1* and *HBA2* genes, including cis-acting sequence motifs, such as a TATA box (around minus 30 bp), a CCAAT box (between minus 70–80 bp), and CCGCCC motifs lying further upstream [2]. The *HBA1* and *HBA2* genes are located within the so-called α-globin gene cluster, which spans about 30 kb, containing one embryonic ζ globin gene (*HBZ*), three globin pseudogenes (ψζ_1_, ψα_2_, ψα_1_), and the θ globin gene (*HBQ1*) of undetermined function. Subsequent studies have shown that the ψα_2_ gene, in fact, expresses a transcript named μ-globin (mu-globin), and was thus renamed *HBM* [6]. The genes in the cluster are arranged along the chromosome in the following order: telomere (5′), *HBZ*, *ψζ*_1_, *HBM*, *ψα1*, *HBA2*, *HBA1*, *HBQ1*, and centromere (3′) (Figure 1).

There are four highly conserved, non-coding sequences, known as multispecies conserved sequences (MCSs) located between 10–40 kb upstream (5′) to *HBZ*, named MCS-R1–4, corresponding to previously identified erythroid-specific DNAse1 hypersensitive sites (DHSs) referred to as HS-48, HS-40, HS-33, and HS-10, whereby the number refers to the distance in kilobases from the *HBZ* gene CAP site. Evidence indicates that only the presence of HS-40 is essential for the expression of *HBA1* and *HBA2*, and the role of the other three MCS elements remain undefined to date (Figure 1) [6].

### 3.1. Normal Expression of Alpha-Globin Genes

The *HBZ* gene is expressed until about the 6th week of gestation in humans, and both α- and ζ-globins are observed in the primitive erythroblasts which, until this stage of development, originate from the yolk sac. Studies in mice have shown that, as such cells mature, there is a switch from ζ- to α-globin expression, and it is likely that there is a similar switch in humans. Following the 6th week of gestation, the erythroblasts originating from the liver almost exclusively synthesize α-globin. However, minute quantities of ζ-mRNA are still produced, throughout fetal life, and form Hb Portland (ζ2γ2), detected in the cord bloods of non-thalassemic newborns. Very low levels of θ-globin mRNA transcripts from *HBQ1* have been detected in yolk sac, fetal liver, adult blood, and bone marrow and similarly, very low levels of μ-globin mRNA can be detected in cord blood, adult blood, and bone marrow, but neither have yet been observed at the protein level [6,8].

The transcription of the *HBA1* and *HBA2* genes during hematopoiesis begins at the stage of basophilic erythroblasts, reaching a peak in the intermediate (polychromatophilic) erythroblasts, but subsequently stops as the erythroid cell matures. However, even following the extrusion of the nucleus, sufficient levels of globin mRNA persist in reticulocytes, such that globin polypeptide chains continue to be synthesized for a further 2–3 days, facilitated by the long half-life (>24 h) of globin mRNA. The protein products of the *HBA1* and *HBA2* genes are identical, although in fetal and adult life there is approximately three times as much α2-mRNA compared to α1-mRNA in erythrocytes from normal individuals. Normal erythroblasts express four (4) α-globin genes [8].

### 3.2. Genomic Variants in the Alpha-Globin Gene Cluster

Over 380 variants involving the α-globin genes (*HBA1*, *HBA2*) and wider clusters have been associated with α-thalassemia to date (see the Ithanet database at https://www.ithanet.eu (accessed on 24 January 2024) and the HbVar database at http://globin.cse.psu.edu (accessed on 24 January 2024)). As for most variants associated with hemoglobinopathies, many are population-specific, although recent genotyping technologies have identified many sporadic novel variants. The majority of individuals with α-thalassemia have genotypes involving deletion variants that remove some, or all of, the α-globin gene cluster. Less commonly, the defects are single or oligonucleotide variants or very small deletions within either of the duplicated *HBA1* or *HBA2* genes. Variants which partially abolish the synthesis of α-globin chains by the affected chromosome are traditionally termed α^+^ thalassemia variants, and those that completely abolish the synthesis of α-globin (null variants) are termed α^0^ thalassemia variants [9,10].

The category of variants most frequently observed in individuals with α-thalassemia are deletions which leave a single functional α globin gene on the chromosome. The two most common α^+^-thalassemia deletions include one of 3.7 kb or of 4.2 kb, both arising from unequal cross-over during meiosis, due to highly homologous sequences surrounding the *HBA1* and *HBA2* genes (Figure 1). The reciprocal chromosome causing the deletion of a single α-globin gene is an allele with three α-globin genes, often termed a “triplicated” arrangement (ααα^anti3.7^ and ααα^anti4.2^, respectively). There are over 250 million carriers of α^+^- thalassemia in the world, with the highest incidence found in the populations of India, Southeast Asia, and Africa, and less commonly in the Mediterranean and Middle East [9].

Most variants causing α^0^ thalassemia remove at least the two α-globin genes from the affected chromosome, and in some cases the complete ζ–α-globin gene cluster. In addition, several deletions have been characterized that leave the α-globin genes intact but remove conserved regulatory elements, including the critical HS-40, along with variable amounts of flanking DNA. With such deletions, the α-globin genes, although present, are not expressed, giving rise to a phenotype of α-thalassemia. Most α^0^ thalassemia deletion variants are sporadic. However, four are more common, with so-called --^Med^ and (−α)^20.5^ found in Mediterranean populations, and --^SEA^ and –^FIL^ in Southeast Asia [9].

More than 250 single nucleotide variants (substitutions, microdeletions, or microduplications) relate to α-thalassemia and are more often observed in *HBA2* gene than *HBA1*. These variants disrupt all the known stages of protein synthesis (including correct RNA processing or translation). Additionally, an interesting group of single nucleotide variants lead to the synthesis of highly unstable α-globin polypeptides, which mimic an α-thalassemia phenotype through reducing the production of normal α-globin chains. These variants are rarely detectable at the protein level (in the form of hemoglobin) but, as they are usually detected through genotyping methods, the amino substitution can be predicted and the variants are often named as hemoglobins, e.g. Hb Quong Sze (α2 cd125 CTG > CCG; Leu > Pro), Hb Agrinio (α2 cd29 CTG > CCG; Leu > Pro), and Hb Taybee (α1 cd38 or 39 delACC; Thr) [9].

## 4. The Impact of Changes in the Expression of Alpha-Globin Genes on Hematopoietic Pathologies: Genetic Evidence

In populations with a high frequency of α- and β-thalassemic variants, the incidence of complex genotypes, due to the co-inheritance of α- and β-thalassemia, is not rare. These genotypes, with the associated hematological and clinical phenotypes, are useful to illustrate the deleterious effect of the excess of free α-globin chain monomers, the relative level of which is the major factor contributing to the severity of β-thalassemia syndromes [11,12,13]. In β-thalassemia heterozygotes that coinherit less than four α-globin genes (heterozygous α-thalassemia genotypes), diminished levels of excess α-globin chains are expected. Comparison of the hemoglobin levels and red cell indices between the various groups with three or two functional α-genes (-α/αα, --/αα, or -α/-α) with heterozygous β-thalassemia demonstrates a relative increase in hemoglobin levels and red cell indices (MCV and MCH), compared to those of β-thalassemia heterozygotes with the normal number of four functional α-globin genes. The amelioration of these parameters was most apparent in cases with two α-genes deleted, since there is a balanced/almost balanced α/β chain ratio. However, in cases with heterozygous β-thalassemia and only one functional α-globin gene, patients have chronic anemia analogous to that found in patients with hemoglobinopathy H (HbH disease), but without detectable levels of HbH [13].

There is evidence that the co-inheritance of α-thalassemia genotypes which reduce α-globin synthesis may modify the clinical phenotype of homozygous or compound heterozygous β-thalassemia to a milder form, compatible to NTDT. This effect is more obvious in cases with *HBB* genotypes that allow some expression of β-globin (so-called β^+^) compared to null variants (so-called β^0^) [13].

When cases with homozygous or compound heterozygous β-thalassemia alleles co-inherit only a single functional α-globin gene (a genotype typically associated with HbH disease), the rare cases described have a phenotype of non-transfusion-dependent thalassemia (NTDT) rather than transfusion-dependent thalassemia (TDT) [13,14,15]. The male patient described by Kanavakis et al. [13] was first hospitalized at 50 years of age due to fatigue. Examination revealed marked anemia (hematocrit 20%), though he had never been transfused. Following splenectomy, he maintained hemoglobin levels of 85–95 g/dL. DNA analysis identified *HBB* genotypes equivalent to TDT (β^+^/β^0^), with *HBA1/2* alleles involving a single α-globin gene in-trans to complete the deletion of both α-globin genes, equivalent to HbH disease (α^+^/α^0^). The α/non α-globin chain biosynthesis was completely balanced. Hematological parameters demonstrated a well-compensated anemia with ineffective erythropoiesis and oxidative stress, which was ameliorated following splenectomy [13].

In contrast, alleles with additional α-globin genes, most commonly triplicated α-globin gene arrangements, have been observed to co-inherit with heterozygous β-thalassemia genotypes. The majority of the studies evaluating hematological and phenotypic characteristics in β-thalassemia heterozygotes with a single additional functional α-globin gene report that the additional α-globin gene had a marked effect on the hematological phenotype in most cases and often also on the clinical phenotype. Specifically, double heterozygotes present lower hemoglobin levels and red cell indices, and a clinical presentation is often categorized as NTDT with occasional (and exceptionally more regular) transfusion requirements [16,17,18,19,20,21,22]. In contrast, some publications concluded that an additional α-globin gene had a very limited effect on the hematological phenotype of β-thalassemia heterozygotes [23], or even no significant effect [24], with the latter presenting the largest cohort described so far, including 67 cases.

These contradictory results may be attributed to differences in the strategies used to recruit the cases in each study, as well as the criteria for the evaluation of hematological and clinical phenotypes (summarized in Supplementary Table S1). The cohorts that were selected based on a phenotype-driven strategy tended to conclude that the additional α-globin gene exacerbated the phenotype of heterozygous β-thalassemia. In contrast, the studies of cohorts collated based on chance identification of the double heterozygous genotype (e.g., following routine genetic analysis or carrier screening), found no significant consequence. In conclusion, not every double heterozygote has a significantly affected phenotype, indicating the likely contribution of numerous other genetic parameters, as well as environmental and socioeconomic factors.

Despite the differences regarding the effect of five α-globin genes in β-thalassemia heterozygotes, there is an agreement that the interaction of six active α-genes, especially when co-inherited with heterozygous β^0^-thalassemia, almost always, produces NTDT [16,25,26,27,28,29,30]. These six functional α-globin genes could be the result of homozygosity for the so-called anti 3.7 kb or anti 4.2 kb arrangements or, rarely, of heterozygosity for quadruplicated α-globin gene arrangements [26,30,31], while the presence of more than six functional α-globin genes (i.e., compound heterozygosity ααα/αααα) has been associated with TDT and NTDT, the latter with a relatively more severe presentation [30,32]. An interesting example is reported by Farashi et al. in 2015 [32], regarding a family in which both the father (at the age of 30) and his daughter (at the age of 6 months) required blood transfusions, bimonthly for the father and monthly for the child. Both father and daughter were heterozygous for a β^+^-thalassemia variant. The mother, a hematologically asymptomatic subject, was found to carry a duplication analogous to anti 3.7 kb, while the father carried an extra complete α-globin gene cluster, resulting in four functional α-globin genes from this allele (total six α-genes), whereby the child was found to be a compound heterozygote with seven α-globin genes [32].

Overall, these studies indicate that the levels of excess α-globin chains in the erythrocytes are a major factor modifying the severity of beta-thalassemia syndromes [33].

## 5. The Impact of Changes in the Expression of Alpha-Globin Genes on Hematopoietic Pathologies: Evidence in the “Gene Editing Era”

**Figure 2:**
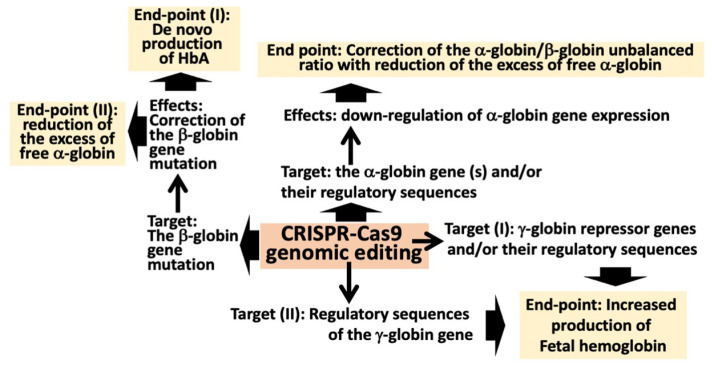
Applications of CRISPR-Cas9 gene editing to β-thalassemia for correction of β-globin gene variants, reduction in the excess of free α-globin, and increased production of fetal heoglobin (HbF).

The clinical relevance of the inbalanced expression of α-globin versus β-globin genes is strongly in agreement with data obtained by genome editing approaches, as is summarized in Figure 2.

Genome editing (GE) can be considered among the most promising strategies to correct hereditary alterations in a variety of monogenic diseases, including hematopoietic pathologies [34,35]. Figure 2 depicts how CRISPR-Cas9 gene editing can be applied to β-thalassemia [34,36,37,38,39,40,41,42,43,44]. CRISPR-Cas9 gene editing can be proposed for efficient correction of the NM_000518.5:c.118C>T thalassemia variant (HGVS nomenclature, β^0^39C>T traditional nomenclature) [45]. In addition, CRISPR-Cas9 gene editing has been applied for the reduction of the content of free α-globin chains [46,47,48,49]. With the objective of decreasing α-globin gene expression, Mettananda et al. [49] described the use of CRISPR-Cas9 genome editing to mimic a natural mutation, which deletes the MCS-R2 α-globin enhancer and causes α-thalassemia. When edited CD34^+^ cells from β-thalassemia patients were differentiated into erythroid cells, they observed the expected reduction in α-globin expression and a correction of the α/β globin chain imbalance, suggesting that this CRISPR-Cas9 based approach might be of clinical relevance [49]. A second study on this very important issue was published by Pavani et al. [48], demonstrating the correction of the pathological phenotype of β-thalassemia by CRISPR-Cas9 editing of the α-globin locus in human hematopoietic stem cells.

## 6. Alpha-Hemoglobin-Stabilizing Protein (AHSP): Expression, Function, and Molecular Genetics

Alpha-hemoglobin-stabilizing protein (AHSP) is a molecular chaperon with high cell-type specificity, which reversibly binds to free α-globin polypeptides. This binding supports the stability, folding, and assembly of α-globin chains. Additionally, AHSP can promote the refolding of denatured α-globin polypeptide chains. Overall, AHSP prevents the precipitation of free α-globin chains, and when β-globin polypeptide chains are present, it facilitates the formation of tetrameric HbA molecules. In the absence of AHSP, the α-globin polypeptides generate reactive oxygen species (ROS) and precipitate within the precursor erythrocytes in the bone marrow, prompting apoptosis and ineffective erythropoiesis. The *AHSP* gene is located on human chromosome 16 (Chr16p11.2). It is approximately 1.8 kb long, has three exons and two introns, and encodes a polypeptide of 102 amino acids. The AHSP polypeptide forms a bundle of three antiparallel α-helices, whereby helices 1 and 2, and the intervening segment, recognize the G and H helices of the α-globin chains (the latter involved in the α1β1 contact in the HbA molecule) to form a simple heterodimer. The AHSP and β-globin polypeptides compete to bind to α-globin polypeptides within the same region of the protein, but the intermolecular contacts between α-globin chains and AHSP are less extensive than those between α-globin and β-globin chains and, thus, the β-globin chains displace AHSP and form HbA molecules. AHSP can bind α-globin chains with heme or without heme (holo-*α*Hb or apo-*α*Hb, respectively) in both reduced (ferrous, FeII) and oxidized (ferric, FeIII) states. The ASHP protein cannot bind to β-globin polypeptide chains or to tetrameric HbA molecules. AHSP is expressed exclusively in hematopoietic tissues at high levels [50,51]. The promoter of the *AHSP* gene extends from the 5′-flanking region until intron 1 (Base pairs −170 to +269) and includes five consensus GATA-1 binding sites, an Oct-1 consensus site, and an EKLF binding site [52,53]. Therefore, transcription factors GATA-1, Oct-1, and EKLF are expected to coregulate *AHSP* gene expression during erythropoiesis [50,51]. 

Several studies have demonstrated a correlation of AHSP and α-globin expression in erythroblasts from normal individuals and also with β^0^ thalassemia [54], and there may be several potential pathways involved in the upregulation of AHSP expression in the presence of higher levels of free α-globin chains. These mechanisms may not be exclusive and include a feedback mechanism of excess α-globin levels [55], a response to increased ROS induced by excess α-globin chains, as mediated by Nrf2 (nuclear factor erythroid 2-related factor 2) and possibly other components of ROS signaling pathways [56], and a mechanism through iron levels enabled by iron responsive elements and iron regulatory proteins [57].

A few studies have investigated the genetic variation in and around the *ASHP* gene locus across several populations. Based on single nucleotide polymorphisms (SNPs) and a single nucleotide repeat, 18 different *AHSP* gene haplotypes have been determined [58,59]. With respect to genomic variation that may potentially alter the expression of the AHSP protein, a common SNP (G>A) at position 12391 within intron 1, was found to disrupt a proposed binding site for transcription factor Oct-1, potentially inhibiting the optimum activation of AHSP gene expression [59]. 

Missense variants causing altered protein structure are rare in all populations studied (see below), but there are several examples reported. The substitution of Asn75 > Ile (N75I), although it is not located in a region of the AHSP polypeptide important for forming a complex with α-globin, studies found that AHSP with the Asn75 > Ile variant has impaired ability to inhibit reactive oxygen species (ROS) production by α-globin, potentially explaining the deleterious clinical effect of this mutant [59]. Based on hematological parameters in β-thalassemia heterozygotes in Southern China, the rare variants AHSP Asp29 > Val (D29V) and AHSP Val56 > Gly (V56G) found no apparent modifying effect [60], although another study reported that Val56 > Gly (V56G) may be associated with some instability and an increased dissociation rate of the AHSP/α-globin complex [61]. Ray et al. 2019 reported the Ser33 > Phe (S33F) variant was postulated to disrupt the structural formation of AHSP with a negative result on its normal function [62]. Studies which evaluated AHSP mRNA levels in peripheral blood reticulocytes of healthy individuals observed a three-fold variation between individuals that did not correlate with age or sex [62]. This study found that the level of AHSP depended upon the associations of several *AHSP* haplotypes linking to some clades, possibly associated with sequence variants within the promoter region, including the Oct-1 consensus site [63]. A classical twin heritability study in unselected twins by the same group found that >45% of AHSP expression was influenced by genetic factors, while almost 30% was influenced by environmental factors [64].

In conclusion, although the expression of AHSP has been positively correlated to the levels of excess α-globin in normal and β-thalassemia erythroblasts [54,55], the underlying genetic variability at the ASHP locus which may influence the expression of AHSP has not been completely characterized to date.

## 7. Alpha-Hemoglobin-Stabilizing Protein as a Modifier of Beta-Thalassemia

Based on the evidence that the level of excess α-globin chains is one of the key factors underlying the severity of β-thalassemia phenotypes and clinical course, ASHP has been proposed as a potential candidate for modulating this severity. Furthermore, an AHSP gene knock-out mouse model presents a similar phenotype to the β-thalassemic mouse model [50,65]. Structural variants in the AHSP gene are uncommon [58,59], and are therefore unlikely to be major modifiers of β-thalassemia in most populations, although the influence of rare AHSP null or missense variants, such as the rare coding AHSP N75I variant, may impair normal AHSP protein function and, thus, the phenotype of β-thalassemia. The first study to implicate a potential influence of AHSP on modulating β-thalassemia phenotypes found reduced erythrocyte AHSP mRNA expression in β-thalassemia heterozygotes with an unusually severe phenotype of NTDT, compared to phenotypically typical β-thalassemia heterozygotes and normal individuals [66]. Of note is that this study did not investigate DNA variants at the *AHSP* locus or elsewhere in the genome, and that, as mentioned above, mRNA levels were subsequently demonstrated to have significant variability, even between normal (non-thalassemic) individuals [63]. In contrast, another study of Thai patients with HbE/β-thalassemia concluded that there was no correlation of clinical severity with *AHSP* gene variants [58], and similar conclusions were also made in a study of investigating AHSP gene polymorphisms in β-thalassemia heterozygotes from a Southern Chinese population [60]. Another study which measured AHSP mRNA levels in a cohort of 37 β-thalassemia patients found no significant difference in the levels of AHSP between patients with TDT and NTDT, although the levels were found to be higher in 12 sickle cell patients [67]. A more recent study by Ray et al. performed AHSP gene sequencing in samples from 38 patients with HbE/β-thalassemia [62]. Comparing the *AHSP* variants observed in 23 TDT and 15 NTDT patients, there was a statistically significant correlation with three of five variants between the two groups. The three variants with statistical significance included two silent nucleotide substitutions and one missense variant (Ser33 > Phe or S33F), with the latter, which was observed in 65% of patients in the TDT group and postulated to potentially disrupt the normal function of the AHSP protein, observed in 65% of patients in the TDT group.

Overall, it should be underlined that it is yet to be clearly established whether (or not) AHSP plays a role in modifying the phenotypic severity in β-thalassemia. In this respect, it is difficult to drive robust conclusions comparing studies that have so far focused on a variety of heterogenous cohorts of β-thalassemia patients, using different biochemical and molecular strategies to evaluate the effect of AHSP variants on AHSP expression, based on heterogenous protocols for measuring AHSP mRNA and protein levels, or correlating phenotypes of patients and/or heterozygotes with genetic variation at the AHSP locus. In conclusion, although the proposed function of the AHSP protein makes it an attractive therapeutic agent in potentially ameliorating the clinical course in β-thalassemia patients [68], further studies are required to clarify a correlation of β-thalassemia phenotypes with AHSP protein expression.

Finally, it is of interest to note that several α-globin chain variants have been described, which are prone to structural instability associated with structural alterations and impaired interaction with AHSP [69,70,71]. This is the case of the recently described α-globin frameshift mutants with stop codon in the last exon at codon 102 (Hb Campania) or at codon 133 (Hb Sciacca) [71]. A 3D model indicated instability of the α-globin chain variants, due to the severe structural alterations causing an impairment of the molecular interactions with AHSP. Similarly, Hb Bronovo [α103(G10)His → Leu, HBA2: c.311A>T] is an α-globin variant that interferes with the AHSP’s binding efficiency. In fact, the histidine residue at position 103 is integral to the AHSP’s hydrogen bond formation and its disruption leads to an increased quantity of cytotoxic free α-globin chains, thereby creating a similar pathophysiology as β-thalassemia [69].

## 8. Alpha-Hemoglobin-Stabilizing Protein as a Modifier of Sickle Cell Disease

There is little evidence about the influence of AHSP on sickle cell disease (SCD). The expression of the *AHSP* gene has been reported to be higher in SCD erythrocytes, with respect to unaffected controls. Mahmoud et al. (2015) found that median AHSP expression was significantly higher in patients with SCD, compared to thalassemia patients [67]. Vasseur et al. (2022) were able to demonstrate higher AHSP content in red blood cells of SCD patients with or without hydroxyurea (HU) treatment [72]. The interplay between expression of α-globin genes, the presence of free α-globin, the production of AHSP, and the phenotype of sickle cell disease (SCD) has not been evaluated in as much in depth as for β-thalassemia. However, some studies have been reported, suggesting that this field of investigation deserves consideration. For instance, a limited number of relevant studies demonstrate the impact of genetic variations in α-globin genes on SCD severity.

### 8.1. Interaction of Alpha-Thalassemia and Homozygous Sickle Cell Disease

The interaction of α-thalassemia and homozygous SCD were reported in different studies [73,74,75] concurrently showing that α-thalassemia reduces the hemolytic rate in homozygous SCD patients [75]. In agreement with these reports, Kirkham et al. have recently reported that α-thalassemia deletions in SCD patients are significantly associated with improvements in clinical and biochemical parameters, such as increased hemoglobin levels and a reduced risk of albuminuria, abnormal transcranial Doppler velocity, and stroke [76]. Interestingly, a single alpha-globin gene deletion, referred to as the alpha thalassemia silent carrier, is present in more than 30 percent of SCD patients of African descent, with an even higher prevalence in some SCD populations in the Middle East and India [77]. A second issue is related to the presence of excess free α-globin chains in SCD. Vasseur et al. reported an elevated soluble α-hemoglobin pool in sickle cell anemia [78]. This finding was confirmed by Domingues-Hamdi et al., who also demonstrated that hydroxycarbamide decreases this free α-hemoglobin pool in red blood cells of adult patients with SCD [79].

### 8.2. Levels of Alpha-Hemoglobin-Stabilizing Protein in Sickle Cell Disease

The levels of AHSP in red blood cell lysates from patients with SCD were analyzed by different research groups, to evaluate the clinical relevance of *AHSP* gene expression [67,72]. The first information that Vasseur et al. [72] obtained was that the AHSP concentration was significantly higher in patients with the SS genotype than in the controls. The second observation was that a strong positive correlation was observed between the AHSP concentration and the α-hemoglobin pool. This is of relevance, considering that the free α-Hb pool of patients with SCD is higher than that of controls. Therefore, it is likely to hypothesize that the AHSP concentration increased in these patients to compensate for the relative excess of free α-globin chains [67,72]. In conclusion, even though the real benefits of AHSP expression on SCD phenotype have not been conclusively demonstrated, some interesting reports are available, suggesting that the impact of AHSP on SCD should be still considered in future research efforts.

## 9. Role of Alpha-Hemoglobin-Stabilizing Protein in Normal and Pathological Erythropoiesis: Updates from Studies Based on Transgenic Mouse Model Systems

Transgenic mice have been extensively used to study the role of AHSP and propose strategies for ameliorating the clinical phenotype of β-thalassemia. In a first study, Kong et al. generated *AHSP*^–/–^ mice by gene targeting [65], demonstrating an abnormal erythrocyte morphology with hemoglobin precipitates in these animals. Furthermore, they found that the loss of AHSP reduced the lifespan of circulating red-blood cells (RBCs) and increased ROS-dependent apoptosis of erythroid precursors [65]. Importantly, when informative interbreeding of mutant mice was performed, it was found that loss of AHSP exacerbated the severity of β-thalassemia. In agreement, Wang et al. [80] employed a human AHSP vector to generate transgenic human AHSP mice in a model of “β^IVS-2-654^-thalassemia”. AHSP expression was associated with improvement in the red blood cell parameters. For instance, a dramatic reduction in anisocytosis in the peripheral blood was observed. Splenomegaly with extramedullary hematopoiesis was ameliorated. Serum iron concentration and iron deposition in the liver were decreased in “h-ahsp^+^/βIVS-2-654^+^” mice. All these findings suggested amelioration of the anemia phenotype in “h-ahsp^+^/β^IVS-2-654^” mice after the introduction of the *AHSP* gene. They therefore propose that an *AHSP* transgene could provide an adjuvant method for gene therapy of β-thalassemia. However, other mice model systems have been described, in which AHSP does not limit α-globin detoxification. In this context, Nasimuzzaman et al. used transgenic mice to investigate the effects of supraphysiologic levels of AHSP on the severity of a NTDT β-thalassemia phenotype [81]. They tested wild-type AHSP and two mutant versions exhibiting 3- or 13-fold higher affinity for α-globin. Erythroid overexpression of these AHSP proteins, up to 11-fold beyond endogenous levels, had no major effects on hematologic parameters in β-thalassemic animals. Therefore, while a consensus does exist on the exacerbation of the β-thalassemia related parameters in the case of low (or absent) AHSP production [65,82], supraphysiologic levels of AHSP might not be associated with clinical improvements [81]. On the other hand, a partial improvement in erythroid parameters has been proposed by Wang et al., associated with AHSP production [80]. This specific issue needs further investigations to associate the endogenous levels of AHSP with the β-thalassemia phenotype in patients with different β-thalassemia genotypes and endogenous levels of AHSP.

In conclusion, AHSP activation could be beneficial, to reduce the physio-pathologic alterations caused by the precipitation of α-globin aggregates in erythroid β-thalassemic cells. Strategies to achieve higher AHSP levels can be designed by taking advantages from studies on the molecular regulation of *AHSP* gene expression. For instance, the direct binding of STAT3 to the *AHSP* promoter is involved in the upregulation of the *AHSP* gene, providing clues to therapeutic strategy for *AHSP* enhancement [52,53]. 

## 10. Inducers of Alpha-Hemoglobin-Stabilizing Protein

Given the recognized importance of AHSP for the lifespan of erythroid cells, AHSP inducers might be of interest, from a therapeutic point of view. In respect to this issue, Liu et al. [83] found that nitidine chloride (NC) induces erythroid differentiation of human leukemic K562 cells, together with increased expression of the genes coding the α-, ε-, and γ-globins, and the erythroid differentiation markers AHSP, CD235a, and CD71. It should be of interest to verify whether NC is able to induce increased expression of AHSP in erythroid precursor cells from patients with β-thalassemia, especially those exhibiting an excess of free α-globin chains. In a more recent study, Han et al. demonstrated that AHSP expression in K562 cells can be stimulated by NFE2-related factor 2 (Nrf2) and its agonist tert-Butylhydroquinone (tBHQ) [56]. Interestingly, the AHSP levels were elevated in α-globin-overexpressing K562 cells and erythroblasts from β^IVS-2-654^ thalassemic mice. In these experimental model systems, tBHQ treatment partially alleviated, whereas Nrf2 or AHSP knockdown exacerbated, α-globin precipitation and ROS production in fetal liver-derived thalassemic erythroid cells. In this context, it should be mentioned that sulforaphane (SFN), a naturally occurring isothiocyanate found in cruciferous vegetables, has received attention as a natural activator of the Nrf2/Keap1 cytoprotective pathway [84]. Other NRF2 activators have been reported and recently reviewed [85,86]. In the search for AHSP inducers, it is relevant to consider that, in erythroid cells, *AHSP* gene expression is facilitated by several transcription factors, such as GATA1, EKLF, OCT1, and STAT3 [87,88,89]. In contrast, a reduction in AHSP synthesis in hemin-induced K562 cells leads to alpha-globin precipitation, the impairment of hemoglobin production, and increased cell death [90].

## 11. The Clearance of Free Alpha-Globin Is Activated by Autophagy in Erythroid Cells from Beta-Thalassemia Patients

**Figure 3:**
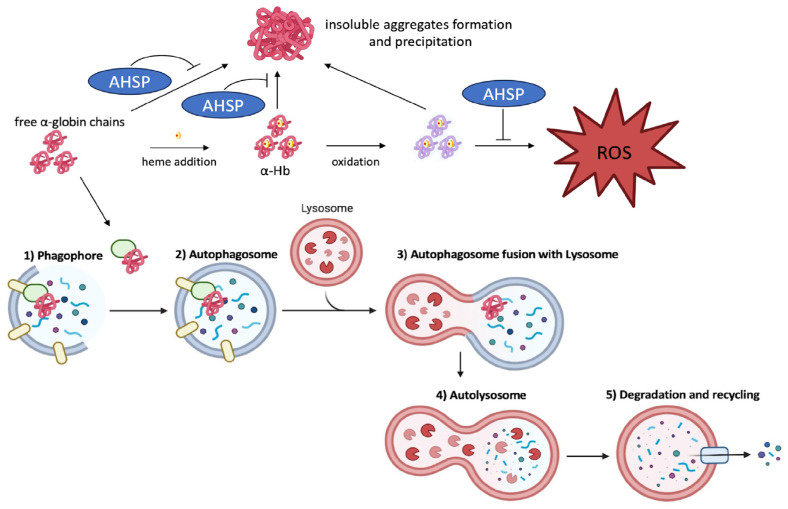
Autophagy and AHSP cooperate to reduce cellular damage induced by toxic α-globin accumulation in β-thalassemia. AHSP prevents precipitation of α-globin chains and the formation of ROS; on the other hand, autophagy recruits free α-globin chains accumulating in erythroid cells through p62 or other cargo protein interaction for transport into the autophagic vesicles. Once autophagosomes with waste material are formed, they undergo fusion with lysosomes to form autolysosomes; finally, the cargo is degraded by acidic hydrolases and resulting nutrients are made available again to the cell.

Autophagy, a highly conserved process of degradation of cellular components among mammals, plays a crucial role in the recycling of cytosol and organelle-derived macromolecules. This process involves the formation of autophagosomes, which are double membrane vesicles where the macromolecules to be degraded are encapsulated [91]. Subsequently, these autophagosomes fuse with lysosomes and the waste material is degraded, to be recycled by the cells for other purposes [92]. Regarding erythroid maturation, autophagy plays a significant role by facilitating the clearance of redundant organelles, including ribosomes and mitochondria (referred to as mitophagy). Several studies have demonstrated that this process is crucial for ensuring the proper development of mature red blood cells from erythroid precursor cells isolated from healthy donors [93,94,95,96,97,98,99]. Following this consideration, the autophagic process should be particularly relevant in erythroid precursor cells isolated from β-thalassemia patients, since hemolysis and ineffective erythropoiesis stimulate the bone marrow to increase the production of erythroid precursors and their release into the blood stream. Despite this, few scientific studies have evaluated the autophagy process in depth in β-thalassemia patients [100,101,102,103]. Besides contributing to the maturation of the erythroid cells themselves, an additional role for autophagy in β-thalassemia is to promote the clearance of toxic α-globin excess accumulating in erythroid precursors from β-thalassemia patients [96]. Research by several groups indicates that free α-globin is degraded by autophagy in β-thalassemia [101,104,105]. Therefore, autophagy might be considered a mechanism, in addition to that involving AHSP, by which β-thalassemia erythroid cells are able to mitigate the effects of the excess α-globin production. In addition, autophagy pathways have a clear role in the protection against metabolic and proteotoxic stresses [106], such as those exacerbated by the excess of α-globin in β-thalassemia erythroid cells. In a recent study, Lechauve et al. demonstrated that the absence of the autophagy-activating Unc-51–like kinase 1 (ULK1) gene in a β-thalassemia mouse model impairs the autophagic clearance of α-globin within red blood cell precursors, leading to the aggravation of disease symptoms [100]. Treatment with the mTORC1 inhibitor sirolimus (rapamycin) systemically reduces the accumulation of α-globin aggregates and alleviates the pathological manifestations in β-thalassemic mice [100]. Interestingly, K562 cellular clones forced to express α-globin protein at high levels seem to activate the autophagy process as a defense mechanism [107]. In these cellular clones producing high levels of toxic α-globin, ULK1 mRNA was found to be upregulated, and the number of autophagosomes increased proportionally to the accumulation of α-globin protein, supporting the role of autophagy in detoxifying cells from toxic protein accumulation. In this regard, the mTOR inhibitor sirolimus (rapamycin) is a very interesting molecule that can contribute to the reduction of the content of free α-globin chains in erythroid precursor cells of β-thalassemia patients with two mechanisms: the induction of γ-globin chains with subsequent formation of α_2_γ_2_ tetramers (HbF) [108,109] and the triggering of the autophagy process to enhance the clearance of toxic free α-globin [100]. Zurlo et al. demonstrated that sirolimus induces autophagy in erythroid cells isolated from β-thalassemia patients [106]. The reduction in soluble α-globin chains found in sirolimus-treated erythroid precursors has, moreover, contributed to the moderate accumulation of insoluble α-globin aggregates, responsible for hemolysis in these patients. The interplay between expression and function of AHSP and autophagy is summarized in Figure 3.

Notably, a reduction in free α-globin and the upregulation of ULK1 gene expression was also found in erythroid cells isolated from rapamycin-treated β-thalassemia patients during the NCT03877809 clinical trial [109,110]. In addition, clinical parameters related to hemolysis and ineffective erythropoiesis (such as soluble transferrin receptor, ferritin, and bilirubin levels) were significantly reduced during sirolimus administration in these patients, suggesting that autophagy and α-globin clearance are important and effective processes to be considered during clinical trials involving thalassemia patients.

## 12. Conclusions and Future Perspectives

The pathophysiology of β-thalassemia reflects an imbalance between α- and β-globin chains, with an excess of free α-globin chains causing ineffective erythropoiesis and hemolysis [102]. The reduction in free α-globin chains has a clear clinical impact, as suggested from studies demonstrating that, when α-thalassemia is co-inherited with β-thalassemia, excess free α-globin chains are reduced, significantly ameliorating the clinical severity. Robust evidence supports the concept that reduction in the “toxic” excess of free α-globin can be achieved in β-thalassemia erythroid cells either by increasing the expression of the *AHSP* gene or by activation of the autophagic process. A key player of the induction of autophagy in erythroid cells is ULK1 (the unc-51 like autophagy activating kinase 1). It is presently unknown if the co-activation of AHSP and autophagy occur in β-thalassemia erythroid cells, but this will be presumably considered in future studies by research groups working in this very interesting field. Future perspectives include a possible impact of “microRNA therapeutics” on autophagy. Recent studies linking microRNA modulation with ULK1 expression facilitate further investigation in this area as in the study by Keith et al. demonstrating that loss of miR-144/451 alleviates β-thalassemia by stimulating ULK1-mediated autophagy of free α-globin. This finding supports the potential of miRNA targeting for reducing the excess of free α-globin chains in β-thalassemia and other hemoglobinopathies [111].

In summary, in this short review we focused on the impact of α-globin gene expression and α-globin modifiers on the phenotype and clinical severity of β-thalassemia. It was written on behalf of the INHERENT (International Hemoglobinopthy Research Network) project, concordant with the interests of INHERENT, which has a primary aim to study the role of genetic modifiers in hemoglobinopathies through a large, multi-ethnic genome-wide association study (GWAS) [112]. The evidence to date summarized in this review contributes towards the validation of previously reported genetic modifiers and the potential discovery of new ones, related to the degree of globin chain imbalance in erythrocytes, one of the most important factors influencing the phenotypic expression of human hemoglobinopathies.

## Data Availability

Not applicable.

## Supplementary Materials

The following supporting information can be downloaded at: https://www.mdpi.com/article/10.3390/ijms25063400/s1.
