# Supplementary material for: Impact of α-Globin Gene Expression and α-Globin Modifiers on the Phenotype of β-Thalassemia and Other Hemoglobinopathies: Implications for Patient Management
Source: Int J Mol Sci. 2024 Mar 17;25(6):3400. doi: 10.3390/ijms25063400 (PMC10969871; doi:10.3390/ijms25063400)
Supplement: Supplementary file 1 [file ijms-25-03400-s001.zip › ijms-2892604-supplementary.pdf]

Supplementary Table S1: Summary of selected published studies of cases with heterozygous beta-thalassemia and five functional alpha globin genes (clinical phenotypes are referred to as evaluated by authors in each article)

	Mode of case recruitment					
	Phenotype driven	Genotype identified retrospectively	Number of cases	Hematological Phenotype of cases	Clinical phenotype of cases	Conclusions – effect on hematological and/or clinical phenotype relative to simple β-thalassemia heterozygotes
Traeger-Synodinos et al 1996	✓	✓	11 adults (6 children excluded from this table)	Hb: 10.2±1.1g/dl<br>MCV: 66.7±5.0 fl<br>HbF: 4.9±2.5%<br>Retics: 3.0±1.4%	5/11 with atypical β-thal heterozygotes with moderate anemia and/or mild jaundice	Usually exacerbates hematological phenotype
Camaschella et al 1997	✓	✓	17 cases	**Range of values in:**<br>**Mild cases**<br>Hb: (10g/dl-12.7g/dl)<br>MCV: (59fl-73fl)<br>HbF: (0.9%-5%)<br>**Severe cases**<br>Hb: (6.5g/dl-11.0g/dl)<br>MCV: (60fl-79fl)<br>HbF: (1.5%-13.6%)	6/17 had **mild anemia** with microcytosis and hypochromia<br><br>11/17 **more severe anemia**<br>3/11 had splenomegaly requiring splenectomy<br>4/11 requiring blood transfusions	• Usually exacerbates hematological phenotype<br>• Hematological and clinical variability
Ma et al 2001	✓	✓	8 cases	**Range of values in TI* cases**<br>Hb: (7.3g/dl-9.0g/dl)<br>MCV: (69fl-73.6fl)<br>HbF: (0.7%-19.2%)	2/8 had thalassemia trait<br>6/8 characterized as TI*	• Usually exacerbates hematological phenotype<br>• Hematological and clinical variability
Theodoridou et al 2020		✓	24 cases	**Range of values in**<br>Hb: (6.7g/dl-12,7g/dl)<br>MCV: (55fl-73.6fl)<br>HbF: (0.7%-19.2%)	Great clinical and hematological heterogeneity from asymptomatic to severe TI*.<br>8/14 females transfused sporadically during pregnancy, older age, surgery or cancer.<br>Blood transfusion requirements, apparently associated with underlying HBB genotypes, comorbidities and sex, as based on observation that no male patients in studied group had transfusions, regardless the HBB genotype, while 57.1% of females were transfused, most of them during pregnancy.	• Usually exacerbates hematological phenotype<br>• Hematological and clinical variability
Sundaresan et al 2022		✓	47/61010 referrals (mixed phenotype)	Hb: 9.0±1.9g/dl,<br>MCV: 69.3±6.8<br>HbF: 4.2± 3.2%<br>Retics:2.6.0±1.8%	16/47 (34%) were asymptomatic or minimally symptomatic.<br>30/47 (63,8%) had a NTDT-phenotype.<br>1/47 became transfusion-dependent at 20 years.<br>From 30 cases, with available transfusion history, 12 had required at least one transfusion.<br>From 28 cases with available data 75%, had splenomegaly and 56% had hepatomegaly. Cases with intermediate phenotype had significantly lower Hb and higher HbF levels than asymptomatic ones.	• Usually exacerbates hematological phenotype<br>• Hematological and clinical variability

Ropero et al 2022		✓	64 cases	**Mean &/or range of values in:**<br>**Thalassemia trait**<br>Hb: 12.8g/dl (11.6-13.8)<br>MCV: (58.7fl-68.7fl)<br>HbF: 1.3% (0.4-3.0)<br>Retics: 1.2% (0.6-1.8)<br>**Mild TI***<br>Hb: 10.0g/dl (8.3-11.4)<br>MCV: 64.5fl (55.8-81.3)<br>HbF: 3.7% (0.3-10.1)<br>Retics: 2.7% (0.2-12.0)<br>**Severe TI***<br>Hb: 7.8g/dl (7.2-8.9)<br>MCV: (56.2fl-78.7fl)<br>Retics: 3.1% (1.7-5.1)<br>HbF: 5.2% (0.8-21.7)	9/64 had thalassemia trait<br>39/64 had mild TI*<br>16/64 had severe TI*	• Usually exacerbates hematological phenotype<br>• Hematological and clinical variability
Gurunathan et al 2020	✓		5 non-related individuals with NTDT	**range of values:**<br>Hb: 8.1-10,3g/dl<br>MCV: 54.7-63.7fl<br>HbF: 2.3%- 16%	NTDT**	Exacerbates hematological phenotype<br>α-globin gene copy number is a phenotypic modifier of β-thalassemia
Giordano et al 2009	✓		12/3500 individuals tested	No significant differences compared to β-thalassemia heterozygotes	Typical β-thalassemia trait	No effect observed
Hamid et al 2021	✓		67/4005 β-thalassemia heterozygotes	No significant differences compared to β-thalassemia heterozygotes	Typical β-thalassemia trait	No effect observed

*TI = Thalassemia Intermedia
**NTDT
